# Postoperative Neurocognitive Dysfunction in Patients Undergoing Cardiac Surgery after Remote Ischemic Preconditioning: A Double-Blind Randomized Controlled Pilot Study

**DOI:** 10.1371/journal.pone.0064743

**Published:** 2013-05-31

**Authors:** Patrick Meybohm, Jochen Renner, Ole Broch, Dorothee Caliebe, Martin Albrecht, Jochen Cremer, Nils Haake, Jens Scholz, Kai Zacharowski, Berthold Bein

**Affiliations:** 1 Clinic of Anaesthesiology, Intensive Care Medicine and Pain Therapy, University Hospital Frankfurt, Frankfurt am Main, Germany; 2 Department of Anaesthesiology and Intensive Care Medicine, University Hospital Schleswig-Holstein, Kiel, Germany; 3 Department of Cardiovascular Surgery, University Hospital Schleswig-Holstein, Kiel, Germany; Sapienza University of Rome, Italy

## Abstract

**Background:**

Remote ischemic preconditioning (RIPC) has been shown to enhance the tolerance of remote organs to cope with a subsequent ischemic event. We hypothesized that RIPC reduces postoperative neurocognitive dysfunction (POCD) in patients undergoing complex cardiac surgery.

**Methods:**

We conducted a prospective, randomized, double-blind, controlled trial including 180 adult patients undergoing elective cardiac surgery with cardiopulmonary bypass. Patients were randomized either to RIPC or to control group. Primary endpoint was postoperative neurocognitive dysfunction 5–7 days after surgery assessed by a comprehensive test battery. Cognitive change was assumed if the preoperative to postoperative difference in 2 or more tasks assessing different cognitive domains exceeded more than one SD (1 SD criterion) or if the combined Z score was 1.96 or greater (Z score criterion).

**Results:**

According to 1 SD criterion, 52% of control and 46% of RIPC patients had cognitive deterioration 5–7 days after surgery (p = 0.753). The summarized Z score showed a trend to more cognitive decline in the control group (2.16±5.30) compared to the RIPC group (1.14±4.02; p = 0.228). Three months after surgery, incidence and severity of neurocognitive dysfunction did not differ between control and RIPC. RIPC tended to decrease postoperative troponin T release at both 12 hours [0.60 (0.19–1.94) µg/L vs. 0.48 (0.07–1.84) µg/L] and 24 hours after surgery [0.36 (0.14–1.89) µg/L vs. 0.26 (0.07–0.90) µg/L].

**Conclusions:**

We failed to demonstrate efficacy of a RIPC protocol with respect to incidence and severity of POCD and secondary outcome variables in patients undergoing a wide range of cardiac surgery. Therefore, definitive large-scale multicenter trials are needed.

**Trial Registration:**

ClinicalTrials.gov NCT00877305

## Introduction

Cardiac surgery is associated with a predictable incidence of myocardial, neurologic, and renal ischemia/reperfusion injury. Postoperative neurocognitive dysfunction is also very common in cardiac surgery and is attributable to multiple underlying perioperative factors (e.g., thromboembolism, hypoperfusion, and cerebral inflammation) [1].

Transient sublethal episodes of ischemia in nonvital tissue (e.g., skeletal muscles) have been shown to enhance the tolerance of remote vital organs (e.g., the heart, brain, and kidney) to subsequent prolonged ischemia/reperfusion injury in a number of clinical conditions, a phenomenon known as remote ischemic preconditioning (RIPC). The first proof of principle studies suggested that transient limb ischemia has the potential to attenuate cardiac troponin I or T release during coronary artery surgery [2], [3], congenital heart surgery [4], and noncardiac surgery in high-risk patients [5]. RIPC has now been extended to different organs, representing a general form of interorgan protection against the detrimental effects of acute ischemia/reperfusion injury [6]. This hypothesis is further supported by previous experimental findings suggesting that RIPC also offers advantages with respect to cerebral ischemia/reperfusion injury [7], [8]. Thus, RIPC may represent a simple, noninvasive, and inexpensive procedure for reducing the severity of perioperative ischemic events without any known adverse effects. In the present study, we hypothesized that RIPC reduces the incidence and severity of neurocognitive dysfunction in patients undergoing cardiac surgery with a cardiopulmonary bypass.

### Patients and Methods

This study is a prospective randomized double-blind parallel-group controlled trial examining 180 adult patients undergoing cardiac surgery. All patients received standard perioperative care. No adverse effects have been reported in any of the numerous clinical investigations examining RIPC [2], [3], [4], [5], [9], [10], [11], [12]. The data collection was performed pseudonymously, and the patients’ names did not appear on any case report form or in any other trial document; all collected data were kept confidential. A part of these study data were previously published as an experimental substudy investigating cellular and molecular effects of RIPC in heart tissue [13]. The trial was registered with www.clinicaltrials.gov (identifier: NCT00877305).

### Ethics Statement

The study protocol, patient information, and informed consent were approved by the Ethics Committee of the University Hospital Schleswig-Holstein, Campus Kiel, Germany (Reference number: A165/08). Each patient gave written informed consent to participate in the study. The patients were given enough time and the opportunity to decide whether to participate and to ask any questions before the beginning of study documentation. The study was performed in accordance with the fourth revision of the Declaration of Helsinki (1996). The protocol for this trial and supporting CONSORT checklist are available as supporting information; see Checklist S1 and Protocol S1.

### Inclusion and Exclusion Criteria

After written informed consent was obtained, patients aged ≥18 years who were scheduled to undergo differing types of cardiac surgery in which a cardiopulmonary bypass would be used were included in the study. Thus, the study population examined herein reflected a large group of patients with an increased risk of perioperative morbidity and mortality. The exclusion criteria comprised surgery-related exclusions (off-pump heart surgery, concomitant carotid surgery, minimally invasive surgery with lateral thoracotomy, selective antegrade cerebral perfusion, previous heart surgery, aorta descendens surgery, normothermic cardiopulmonary bypass), cardiac-related exclusions (previous myocardial infarction within the last 7 days; ejection fraction <30%; previous atrial fibrillation within the last 6 months; receiving therapy with amiodarone, digitalis, and other antiarrhythmic agents; having an implanted pacemaker or defibrillator; and emergency cases), previous stroke within the last 2 months, renal failure (defined as a plasma creatinine level of ≥2.0 mg/dL), liver failure, severe alcohol abuse, severe chronic obstructive pulmonary disease, receiving therapy with sulfonamide and nicorandil (which are preconditioning blocking and preconditioning mimetic medications, respectively), previous serious psychiatric disorders (e.g., schizophrenia or dementia), previous serious neurologic illnesses (e.g., Parkinson’s disease), and a Mini-Mental State Examination (MMSE) score <24 points.

### Subject Enrollment

**Figure 1:**
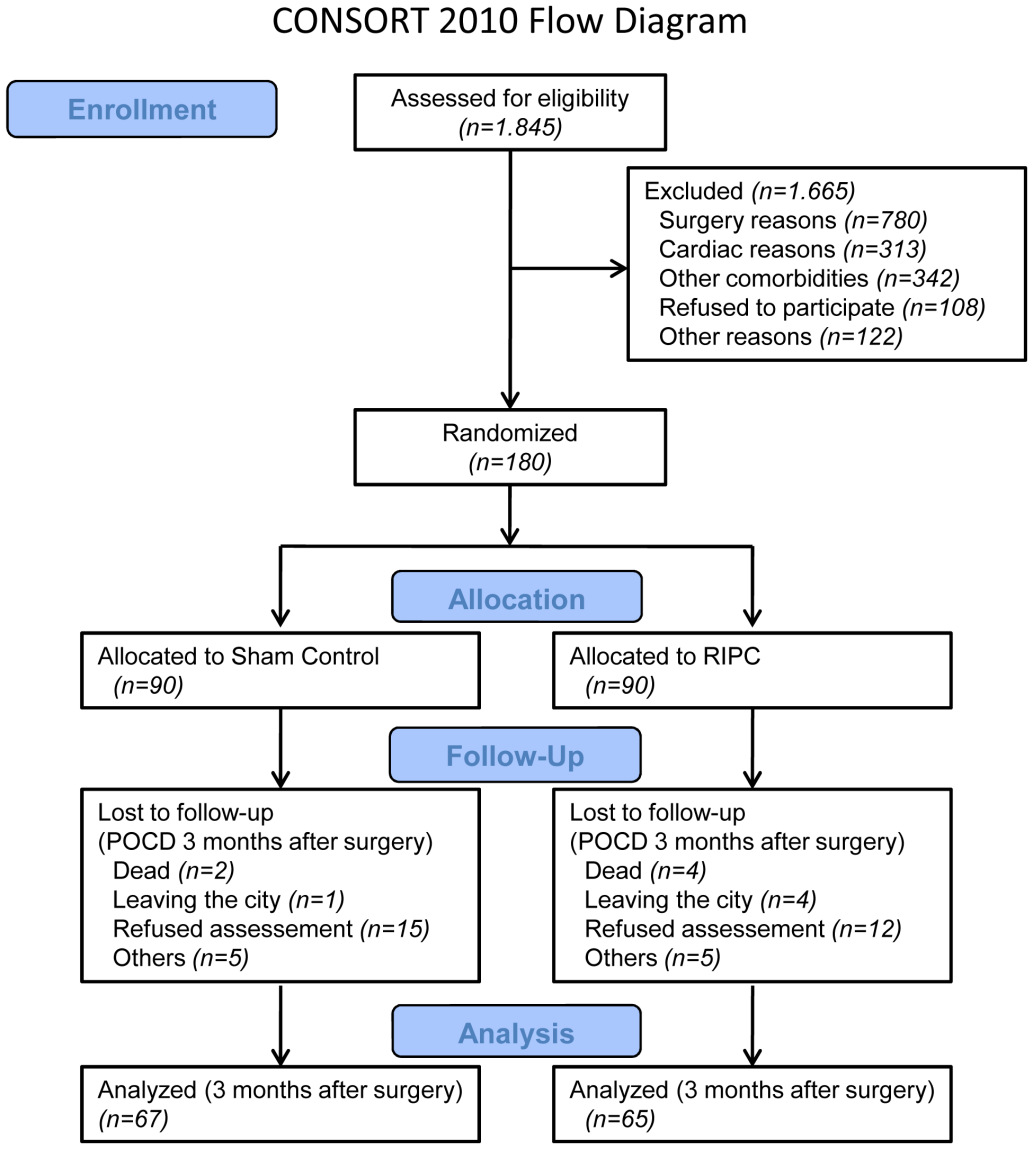
CONSORT flow diagram for individual randomized, controlled trials of nonpharmacologic treatment. Patients scheduled for cardiac surgery with use of cardiopulmonary bypass were included. The day before surgery we checked for eligibility, obtained informed consent, assessed baseline variables, laboratory tests and baseline neurocognitive function. At the day of surgery, standardized general anesthesia and management of cardiopulmonary bypass was performed in all patients. RIPC and control were performed immediately prior to cardiopulmonary bypass. Neurocognitive dysfunction was assessed 5–7 days and 3 months after surgery.

Between January 2009 and November 2010, 1,845 patients were assessed for study eligibility at the University Hospital Schleswig-Holstein, Campus Kiel. In total, 180 patients were enrolled in the study. The participant flow for the study is shown in Figure 1, according to the Consolidated Standards of Reporting Trials group statement [14].

### Intervention

The patients were randomized to either a group undergoing RIPC or a control group. Sealed envelopes were used for randomization. RIPC was induced after induction of anesthesia by 4 cycles of upper limb ischemia (5-min blood pressure cuff inflation to 200 mmHg, a cuff-pressure at least 15 mm Hg higher than the systolic arterial pressure measured via the arterial line, and 5-min cuff deflation). In patients assigned to the sham- control group, we used 4 cycles of 5-min blood pressure cuff inflation to 20 mmHg and 5-min cuff deflation without any limb ischemia. We decided to perform a cuff inflation of the upper limbs to provide continuous access to the blood pressure cuff during the surgical procedure.

The study intervention (RIPC or control) was performed by specially trained staff not involved in the administration of anesthesia and perioperative care and the endpoint assessment. General anesthesia was administered in all patients by an experienced anesthesia team blinded to the group assignment. Standardized perioperative care and management of the cardiopulmonary bypass was provided for all patients blinded to the group allocation. Neurocognitive assessment was performed by a study scientist blinded to the group allocation. Thus, the blinding concerned (1) the individual patient, (2) the staff involved in intraoperative (anesthesia and cardiac surgery team) and perioperative (intensive care unit [ICU] staff) care, and (3) investigators obtaining data, performing the neurocognitive assessment, visiting patients during follow-up, and documenting the study.

### Anesthesia and Management of the Cardiopulmonary Bypass

All patients received standard perioperative care. Total intravenous anesthesia was administered to all patients using propofol (1.5 mg/kg bolus for the induction of anesthesia and 3–6 mg·kg^−1^·h^−1^ continuous infusion during surgery), sufentanil (0.5 µg/kg bolus for the induction of anesthesia and0.5–1.5 µg·kg^−1^·h^−1^ continuous infusion during surgery), and rocuronium (0.6 mg/kg single bolus for tracheal intubation); this anesthetic regimen has been used in routine practice at our institution at the start of the study.

We standardized the management of cardiopulmonary bypass in the trial as follows: a nonpulsatile cardiopulmonary bypass with a membrane oxygenator and cardiotomy suction was used, the target mean arterial blood pressure was 60–70 mmHg, target hematocrit values were 25–30%, patients received alpha-stat management, arterial line filters were used, and cold Buckberg blood cardioplegia solution was used.

The treatment of hypotension, hypertension, bradycardia, and low output was at the discretion of the attending anesthesiologist. However, the recommendations were issued as follows: norepinephrine should be administered for the treatment of hypotension (defined as mean arterial blood pressure <50 mmHg), a glycopyrrolate bolus should be administered for the treatment of bradycardia (heart rate <50 beats per min), and preferably, epinephrine and/or enoximone should be administered for inotropic support. The administration of crystalloid and colloid fluids as well as blood products was allowed to ensure an adequate intravascular volume status. Insulin treatment targeting blood glucose levels <200 mg/dL was recommended.

### Protocol for ICU Sedation and Analgesia

During the initial postoperative period, all patients were sedated with propofol at a dosage of 3–4 mg·kg^−1^·h^−1^ for the first 4–6 h and received a nonsteroidal antiinflammatory drug (e.g., 75 mg of diclofenac) and an intermittent bolus of 0.1 mg/kg piritramid for analgesia. Patients with hemodynamic instability were sedated if the dosage of norepinephrine or epinephrine was >0.4 µg·kg^−1^·min^−1^ or the initial chest tube drainage loss was >150 mL/h. If endotracheal extubation failed within 24 h after surgery, propofol was administered at a dosage of 1–3 mg·kg^−1^·h^−1^ for sedation and piritramid (0.05–0.1 mg/kg) was administered intermittently for analgesia targeting −1 to −2 on the Richmond-Agitation-Sedation-Scale. Propofol was interrupted once daily in the morning. If excessive agitation was noted, propofol was restarted with an additional continuous clonidine infusion (0.5–1 µg·kg^−1^·h^−1^) and an additional piritramid bolus was administered. Propofol was stopped after a maximum of 7 days to reduce the risk of propofol infusion syndrome. If agitation still persisted, patients received midazolam (bolus 0.025–0.5 mg/kg) and piritramid.

### Neurocognitive Assessment

The primary endpoint was postoperative neurocognitive dysfunction 5–7 days after surgery, defined by either the 1-SD criterion (i.e., cognitive change was assumed if the preoperative to postoperative difference in 2 or more tasks assessing different cognitive domains was>1 SD) or the summarized Z score (the combined Z score was ≥1.96). Patients underwent robust neuropsychological tests the day before,5–7 days after, and 3 months after surgery, according to the Statement of Consensus on the Assessment of Neurobehavioral Outcome After Cardiac Surgery [15]. A core battery of 10 tests included the following 4 main domains: memory, motor skills, attention, and executive function (see File S1 for further details on the test assessments).

### Secondary Endpoints

We further determined the duration of ventilatory support, incidence of reintubtion, total length of hospital stay, kidney injury assessed by serum creatinine (the day before, 24 h after, and 48 h after surgery) according to acute kidney injury network (AKIN) criteria [16], myocardial injury assessed by troponin T (the day before surgery; after ICU admission; and 12, 24, and 48 h following surgery), and new onset of atrial fibrillation within 4 days after surgery.

### Statistical Analysis

Statistical analyses were performed using SPSS for Windows, version 20.0 (SPSS, Chicago, Illinois). The values between the groups were compared using 1-way analysis of variance and, in cases of significant differences, adjusted for multiple comparisons using the Bonferroni correction, or the Mann-Whitney U test. The proportions were compared using Fisher’s exact test. Gaussian distribution of each of the neuropsychological test results was examined using the Kolmogorov-Smirnov test. The results of the Rey Auditory Verbal Learning Test with long-term memory, Purdue Pegboard Test of the nondominant and dominant hands, Stroop Color Word Interference Test I–III, Trail Making Test A and B, and Digit Span Test were not normally distributed, and logarithmic transformation was performed to achieve a normal distribution.

We applied 2 definitions of postoperative neurocognitive dysfunction (POCD; the 1-SD criterion and the summarized Z score), which has been used by previous studies investigating cognitive changes after different surgical procedures [17], [18], [19], [20]. According to the 1-SD criterion, a cognitive change was assumed if the preoperative to postoperative difference in 2 or more tasks assessing different cognitive domains was>1 SD. To analyze how many patients of each group were cognitively declined or potentially improved, we calculated the SD of each preoperative test on the basis of the test results from all patients. Because of a lack of a control group, the influence of learning effects on neurocognitive testing could not be analyzed. Therefore, the evaluation of cognitive decline or improvement was limited to a between-group comparison. In a second step, test-specific Z scores were calculated for each patient as the postoperative test result subtracted from the preoperative test result, divided by the preoperative SD of the group. This score indicated the individual change in performance. If appropriate, we changed the algebraic sign so that positive changes indicated deterioration, whereas negative signs reflected improvement. We further calculated the sum of each Z score for all tests and compared the patients in the RIPC group to the - control group using a 1-way analysis of variance. A patient was classified as having POCD if the Z scores on 2 individual tests or the combined Z score was ≥1.96. This definition identified patients with a general deterioration in all tests or substantial deterioration in only some tests [18].

Sample size calculation was estimated in previous studies investigating POCD by Hudetz et al [21] and by our own group [17], [22]. We assumed that the incidence of POCD could be reduced from 30% in the control group to 20% in the RIPC group, and we added 50% of the patients enrolled in the study. For a power of 80% and an alpha error of 5%, we calculated a total of 70 patients in each group. To compensate for approximately 25%of study dropouts, we enrolled 90 patients in each group.

The secondary endpoints were the incidence of POCD 3 months following surgery, myocardial cell damage, atrial fibrillation, incidence of reintubation, acute kidney injury, length of time on a ventilator, and length of hospital stay. These data were analyzed by standard statistical methods for comparing independent samples with respect to their measurement level and type of distribution (parametric and nonparametric methods).

## Results

The demographic data and surgery-related data did not differ significantly between the control and RIPC groups, with the exception that 14 patients in the control group and 30 in the RIPC group had a recent myocardial infarction >7 days ago (*P*<0.05) (Tables 1 and 2). The outcome variables are shown in Table 3. RIPC tended to decrease postoperative troponin T release at both 12 and 24 h after surgery (at 12 h: median,0.60 [range,0.19–1.94 µg/L] for the control group versus median,0.48 [range,0.07–1.84 µg/L] for the RIPC group; and at 24 h: median,0.36 [range,0.14–1.89 µg/L] for the control group versus median,0.26 [range,0.07–0.90 µg/L] for the RIPC group). The incidence of postoperative atrial fibrillation (35 for the control group versus 35 for the RIPC group), endotracheal reintubation (7 in the control group versus 8 in the RIPC group), and renal dysfunction according to the Acute Kidney Injury Network (AKIN) staging system [16] (AKIN stage I: 7 in the control group versus 8 in the RIPC group, and AKIN stage II: 1 in the control group versus 1 in the RIPC group) were comparable between both groups (Table 3).

**Table 1:** Demographic data.

Variable	Control (n = 90)	RIPC (n = 90)
Age, years	68 (23–83)	70 (42–86)
Female gender, n	13	21
**Pre-operative medications**		
Beta-blockers, n	52	65
ACE inhibitors, n	44	37
Long-acting nitrate, n	7	10
Insulin/Metformin, n	9	13
Statins, n	41	52
**Comorbidities**		
Arterial Hypertension, n	73	79
Diabetes mellitus, n	17	21
Recent myocardial infarction, n	14	30*
Preoperative EF, %	67 (30–85)	68 (35–88)
Preoperative creatinine, mg/dL	0.88 (0.48–1.67)	0.82 (0.10–1.93)
Chronic pulmonary disease, n	9	8
Recent stroke, n	4	5
EuroSCORE	3 (0–9)	4 (0–10)

Data are presented as median (range) or absolute number. No difference between groups.

ACE indicates angiotensin converting enzyme; EF, left ventricular ejection fraction.

* p<0.05.

**Table 2:** Type of surgery and surgery-related data.

Variable	Control (n = 90)	RIPC (n = 90)
**Type of Surgery**		
Coronary artery bypass surgery, n	52	54
Number of distal anastomoses, n	3 (1, 6)	3 (1, 7)
Aortic valve replacement, n	12	12
Mitral valve reconstruction, n	1	1
Aorta ascendens replacement, n	4	3
Combined procedures, n	21	20
**Surgery-related data**		
Duration of CPB, min.	121 (46–302)	116 (54–299)
Duration of aortic clamping, min.	80 (32–204)	79 (32–195)

Data are presented as median (range) or absolute number. No difference between groups. CPB indicates cardiopulmonary bypass.

**Table 3:** Outcome data.

Variable	Control (n = 90)	RIPC (n = 90)
Troponin T, µg/L		
Before surgery	0.01 (0.0–0.05)	0.01 (0.0–0.02)
ICU admission	0.45 (0.16–0.96)	0.35 (0.09–1.77)
After 12 hours	0.60 (0.19–1.94)	0.48 (0.07–1.84)
After 24 hours	0.36 (0.14–1.89)	0.26 (0.07–0.90)
After 48 hours	0.26 (0.07–1.57)	0.20 (0.05–0.60)
Atrial fibrillation, n	35	35
Endotrachael reintubation, n	7	8
Renal dysfunction - AKIN I°, n	7	8
Renal dysfunction – AKIN II°, n	1	1
Duration of ventilation, hours	14 (6–696)	14 (6–561)
Total hospital stay, days	9.5 (5–54)	9.5 (5–59)

Data are presented as median (range) or absolute number. No difference between groups. ICU indicates intensive care unit; AKIN, acute kidney injury network.

### Post-hoc Analysis of Patients with Isolated Coronary Artery Bypass Graft Surgery

When focusing on patients with isolated coronary artery bypass graft (CABG) surgery (n = 106), RIPC did not significantly affect postoperative troponin T release (at 24 h: median,0.35 [range,0.14–1.89 µg/L] for the control group versus median, 0.24 [range,0.07–0.60 µg/L] for the RIPC group [*P* = 0.044], and at 48 h: median, 0.18 [range,0.07–1.57 µg/L] for the control group versus median, 0.16 [range,0.05–0.42 µg/L] for the RIPC group [*P*  = 0.275]). After adjustment for multiple comparisons, the small difference after 24 h was no longer statistically significant.

### Neurocognitive Changes 5–7 Days after Surgery Compared with the Baseline Results

All medications that could have influenced neurocognitive functioning were registered. None of our patients were receiving regular doses of opioids preoperatively. Because the exclusion criteria included previous psychiatric disorders and previous neurological illnesses, none of our patients were regularly receiving antidepressants, antiepileptics, or antipsychotic medications. Before initiating POCD testing, a MMSE score of ≥24 points was needed otherwise patients were excluded due to severe cognitive impairment.

Regarding neurocognitive assessment as the primary endpoint of this study, no significant differences in the preoperative test results were detected between the study groups. In total, 56 (31%) of 180 patients did not receive a neurocognitive assessment 5–7 days after surgery (29 patients in the control group and 27 patients in the RIPC group). The reasons for the missing neurocognitive assessment data are shown Table S1 in File S2.

According to the 1-SD criterion, 32 patients in the control group (52%) versus 29 patients in the RIPC group (46%) showed cognitive deterioration in ≥2 tests of different cognitive domains (*P* = 0.753) (see Tables S2 and S3 in File S2). Furthermore, 14 patients in the control group (23%) and 13 patients in the RIPC group (21%) improved their test performance (*P* = 1.0), whereas 6 of these patients in each group showed both a decline in 2 domains and an improvement in the other 2 domains. A comparison of the Z score revealed no significant differences between the groups, although the summarized Z score showed a trend toward more cognitive decline in the control group (mean ± SD: Z score, 2.16±5.30) compared to the RIPC group (1.14±4.02 [*P* = 0.228]) (Table 4).

**Table 4:** Z score in each neuropsychological test 5–7 days and 3 months after surgery compared with preoperative values.

	After 5–7 days		After 3 months	
Domains	Control	RIPC	Control	RIPC
**Memory**				
** RAVLT 1-3**	−0.12 (0.90)	−0.22 (0.97)	−0.41 (0.96)	−0.35 (0.97)
** RAVLT LT**	−0.04 (1.08)	−0.14 (0.78)	−0.15 (0.99)	−0.26 (0.95)
**Motor skills**				
** PBT dominant**	0.65 (0.78)	0.50 (0.89)	−0.16 (0.68)	−0.24 (0.69)
** PBT non- dominant**	0.61 (0.81)	0.53 (0.90)	−0.20 (0.87)	−0.27 (0.85)
**Attention**				
** STROOP I**	−0.34 (0.99)	−0.47 (1.17)	−0.05 (1.13)	−0.07 (1.09)
** STROOP II**	−0.50 (1.15)	−0.65 (0.97)	0.20 (1.06)	0.05 (0.98)
** STROOP III**	−0.20 (0.60)	−0.07 (1.40)	0.24 (0.70)	0.03 (0.96)
** TMT A**	0.09 (0.85)	0.32 (0.83)	0.03 (0.99)	0.24 (0.89)
** TMT B**	0.32 (0.86)	0.13 (1.06)	−0.02 (0.77)	−0.13 (0.69)
** Digit Span**	0.24 (0.85)	0.17 (0.82)	0.01 (0.99)	0.01 (1.00)
** DSST**	0.28 (0.62)	0.20 (0.77)	−0.21 (0.94)	−0.32 (0.75)
**Executive function**				
** VFT semantic**	1.04 (0.98)	0.84 (0.91)	0.21 (1.19)	0.06 (1.23)
** VFT phonetic**	0.34 (1.58)	0.01 (0.94)	0.16 (0.99)	0.00 (1.02)
** Summarized Z score**	2.16 (5.30)	1.14 (4.02)	−0.35 (3.86)	−1.12 (3.70)

Data are presented as mean (SD). No difference between groups. Z score was calculated by subtracting the postoperative test result from the preoperative test result divided through the test specific preoperative SD for each patient. Positive signs indicate deterioration, whereas negative signs reflect improvement.

RIPC indicates remote ischemic preconditioning; RAVLT, rey’s auditorial verbal learning test first to third presentation of words (short-term memory); RAVLT LT, rey’s auditorial verbal learning test long-term memory; PBT dominant, purdue pegboard test performed with preferred hand; PBT non-dominant, purdue pegboard test performed with nonpreferred hand/other hand; STROOP, Stroop color word interference test, first to third run (I-III); TMT, trail making test part A and B; Digit, Digit Span test; DSST, digit symbol substitution test; VFT, verbal fluency test including semantic and phonetic categories (details of the test performance are described in File S1).

### Neurocognitive Changes 3 Months after Surgery Compared with the Baseline Results

Three months following surgery, neurocognitive assessments in 23 patients in the control group and 25 patients in the RIPC group were inconclusive. POCD according to our definition was recognized in 14 patients in the control group (21%; in 7 patients, POCD was also present 5–7 days after surgery) and in 16 patients in the RIPC group (25%; in 5 patients, POCD was already present 5–7 days after surgery). Conversely, 24 control patients (36%) and 20 RIPC patients (31%) showed an improved performance. Of these patients, 1 patient in the control group (1%) and 8 patients in the RIPC group (12%) showed both a decline in 2 domains and an improvement in the other 2 domains. However, neither these differences nor the summarized Z score of all performed tests between the groups was significant (Table 4) (Tables S2 and S3 in File S2).

## Discussion

The first main finding of the present study, with respect to the primary endpoint (incidence and severity of postoperative cognitive dysfunction), was that there was no difference between the patients undergoing RIPC and the control group. The second main finding was that there was also no difference regarding the predefined secondary outcomes such as myocardial cell damage, acute kidney injury, and total length of hospital stay.

### Primary Endpoint

Since the first pivotal animal study by Pryzklenk et al was published [23], the technique of conferring protection to vital organs such as the heart and brain by short and repeated periods of ischemia followed by reperfusion in remote nonvital tissue such as skeletal muscles has attracted attention from both researchers and clinicians. Subsequently, there was growing evidence from animal studies that RIPC (mostly performed as limb ischemia) was able to attenuate ischemic damage in a variety of vital organs [7], [24], [25]. Interestingly, RIPC may also offer beneficial effects with respect to cerebral ischemia/reperfusion injury [8], [26], [27], [28]. Thus, limb preconditioning was recently found to be safe and well tolerated, even at ischemia durations of 10 min, in critically ill patients with subarachnoid hemorrhage [29]. Further, Walsh et al aimed to determine whether RIPC affects neurologic injury following carotid endarterectomy; interestingly, the authors found that there were fewer saccadic latency deteriorations in the RIPC group [30].

In the present study, the summarized Z score showed only a trend to more cognitive decline in the control group compared to the RIPC group 1 week after surgery. Three months after surgery, the incidence and severity of neurocognitive dysfunction did not differ between the groups. At this point, it must be noted that the rate of missing data for POCD testing was higher than we initially estimated. However, as indicated in Table S1, the reasons for missing neurocognitive assessment data were multifactorial. In the majority of patients (n = 30), POCD testing could not be performed because of either prolonged ICU therapy or severe neurocognitive dysfunction with an MMSE score <24 points. In this respect, the high rate of missing data is more the result of the many patients who could not be clinically assessed and less a consequence of a pure loss to follow up. Regarding the missing test results after 3 months, the majority of patients refused to undergo the test battery. Again, the underlying factors were multifactorial, but, in particular, patients with persistent postoperative pain or discomfort, patients with a history of prolonged hospital stay, and patients who experienced negative events during their hospital stay may have tended to refuse any follow-up of clinical trials, thereby biasing the assessment of our study endpoints.

With respect to the underlying pathways of RIPC, we showed that serum taken from patients after RIPC had a different capability of protecting cells during hypoxia than the serum taken before RIPC, and the involvement of matrix-metalloproteinases 2 and 9 in the mechanism conferring protection was demonstrated in the serum taken after RIPC [31]. These data support the idea that humoral factors are involved in RIPC-mediated effects. Further, we previously found in an experimental substudy that RIPC regulated hypoxia-inducible factor1α levels, apoptosis, and inflammation in the myocardium of patients undergoing cardiac surgery and led to increased concentrations of circulating cytokines [13].

### Secondary Endpoints

Recent investigations [2], [4], [9], [32] have fueled the enthusiasm surrounding remote conditioning techniques as a simple, cost-effective intervention without serious side effects while touting these techniques as comparable to the holy grail of perioperative or periprocedural organ protection, and several editorials emphasized the importance of the benefits of remote conditioning techniques in the future [33], [34], [35]. However, the first caution flag regarding RIPC was raised by a neutral trial examining RIPC in patients undergoing cardiac surgery [36]. In this single-center randomized double-blind trial, 162 patients received RIPC or a control intervention. The primary endpoint was the 48-h area under the curve troponin T release and the secondary endpoints, among others, were the cardiac index, use of inotropes and vasoconstrictive agents, and the incidence of renal dysfunction and lung injury. This study did not report any benefit in the predefined primary and secondary outcomes in the RIPC patients. The authors suggested that RIPC applied with the stimulus size, site, and timing in their study failed to confer end-organ protection. It should be noted, however, that volatile anesthetics were used in both groups included in this study. Volatile anesthetics have repeatedly been shown to attenuate myocardial damage in cardiac surgery. It is conceivable that the protective potential inherent in preconditioning was already fully exploited by volatile anesthetic administration and that the additional stimulus of RIPC could not confer further beneficial effects. In a very recent study by Young et al. also examining patients undergoing cardiac surgery, no benefit was seen in the RIPC group compared to the control group with respect to plasma high-sensitivity troponin T, postoperative acute kidney injury based on the RIFLE (risk, injury, failure, loss of kidney function, and end-stage renal failure) criteria, and duration of vasopressor administration [37]. Again, the patients were given volatile anesthetics and had considerably longer bypass times than those reported in previous studies, mainly because complex procedures were allowed for enrollment. Furthermore, >70% of patients in the RIPC group were receiving beta-blockers, a class of agents known to inhibit preconditioning pathways in laboratory investigations [38].

Another explanation for the conflicting results could be that ischemic preconditioning and remote ischemic preconditioning represent a biphasic phenomenon with a first and a second window of protection. The early phase of protection develops quickly within minutes of the initial ischemic conditioning event and lasts for 2–3 h. This is followed by a delayed phase that begins 12 to 24 h after the initial phase and lasts for up to 4 days. In patients with a short intraoperative aortic clamping time, coronary ischemia probably falls into the first window of protection. This may explain why Hausenloy et al. [2] reported beneficial effects of RIPC in patients where cross-clamp times were very short (mean ± SD, 36±17 min in RIPC patients versus 45±22 min in controls). In our study, however, median aortic cross-clamp times were more than twice as long (median, 80 [range, 32–195] min in RIPC patients versus median, 80 [range, 32–204] min in controls). We did not do a post-hoc analysis to evaluate the effect of RIPC in patients with shorter clamping times because only 12 patients (n = 3 for the controls and n = 9 for the RIPC patients) had a cross-clamp time <45 min. In fact, peak troponin T release was significantly correlated with the length of aortic clamping (r = 0.436, *P*<0.001), but not with the type of intervention.

Additionally, significantly more patients had a recent myocardial infarction in the RIPC group that might have further attenuated any preconditioning-associated myocardial protective effect. Finally, preconditioning protocols may have affected the effect size. Most of the previous studies used 3 cycles of 5-min limb ischemia [2], [9], [36]. To further optimize the ischemic stimulus, we used 4 cycles of 5-min upper limb ischemia, which was comparable to other trials [4], [39]. Nevertheless, Ali et al. applied 2 cycles of 10-min ischemia by intermittent cross clamping of the common iliac artery [5]. In this respect, it might be conceivable that preconditioning requires more severe remote ischemia, and this methodologic issue could at least partly explain the lack of an effect in the present study.

When RIPC and volatile anesthetics are combined, the potential benefit of RIPC could be exploited by volatile anesthetics. Contrarily, recent myocardial infarction, beta-blocker administration, or long aortic cross-clamp times may lead to an exhaustion of the protective mechanisms induced by both volatile anesthetic administration and RIPC. Available data from a recent meta-analysis including 15 trials with a total of 1,155 study patients confirmed that cardioprotection was conferred by RIPC in adult patients undergoing cardiac surgery and indicated that the cardioprotective effect may be attenuated when combined with beta-blockers or volatile anesthetics [40].

Despite our study having a different result regarding volatile administration than the study by Young et al, there are some striking similarities [37]. First, our study design was truly double-blind. Second, we comparably enrolled a wide variety of cardiac surgical procedures including complex cases and aortic surgery. Third, a large proportion of our patients were receiving therapy with beta-blockers. Finally, the extent of organ damage was generally low. If we were to analyze the incidence of kidney failure as a sign of organ damage, it appears that only 2 patients presented with AKIN stage II and a total of 15 patients with the AKIN stage I in both groups, which accounts for <10% of the incidence of acute kidney injury in the whole group. This is a very low number compared with the reported incidence in the literature [41].

In specialized centers with a high degree of standardization and a dedicated team of anesthesiologists, surgeons, and perfusionists caring for the patients, a whole bundle of therapeutic measures may decrease the overall incidence of complications, which in turn decreases the visibility of a single protective intervention. Finally, the beneficial effects as indicated in previous trials in isolated CABG surgery with reduced variability of troponin release may be lost if a variety of procedures with variable cross-clamp times and a different extent of direct, procedure-related myocardial damage are present as confounding factors. While we failed to show a beneficial effect with respect to primary and secondary outcomes in our trial, the trend regarding troponin release despite a high proportion of patients receiving beta-blockers and the trend to less severe POCD in the RIPC patients indicate that a definitive answer to the question of whether RIPC is efficacious in a clinical setting is lacking. In this respect, the results of the 2 large-scale multicenter trials underway (ERICCA [NCT01247545] and RIPHeart [NCT01067703] [42]) are eagerly awaited.

### Limitations

Some limitations to our trial should be noted. Because we did not include a control group of patients who did not undergo surgery, the Z score calculation was not based on the decline of such a control group and POCD incidence may have been overestimated in the whole sample. However, this should not have affected the potential between-group differences. As outlined above, beta-blocker therapy may have interfered with the molecular pathways involved in the preconditioning, thereby attenuating possible protection by RIPC; conversely, our trial sought to emulate daily clinical practice as closely as possible. This also holds true for the enrollment of a wide variety of cardiac procedures. For the sake of generalizability, we accepted a high variability of cross-clamp times and procedure-related myocardial (and potentially cerebral) damage. After post-hoc analysis, it was clear that the sample size may have been too small to detect any beneficial effects with respect to our predefined endpoints in such a group of patients. On the basis of our results, future studies should include >150 patients per group to have a chance to detect a significant difference in summarized Z scores, as has been acknowledged in our ongoing multicenter, randomized, controlled trial [42].

In conclusion, we failed to demonstrate the efficacy of a RIPC protocol with respect to the incidence and severity of POCD and secondary outcome variables such as myocardial cell damage, atrial fibrillation, need for reintubation, acute kidney injury, length of time on a ventilator, and length of hospital stay in a relatively small group of patients undergoing a wide variety of cardiac surgical procedures. Because of the relatively small sample size, larger trials are needed for both the primary and secondary end points examined herein.

## Supporting Information

File S1 **Supplemental methods.**
(DOCX)

File S2 **Supplementary tables.** Table S1, Reasons for missing neurocognitive assessment data. Table S2, Results of neurocognitive function assessed before, 5–7 days, and 3 months after surgery. Table S3, Neurocognitive decline/improvement in each neuropsychological test 5–7 days and 3 months after surgery compared with preoperative values.
(DOCX)

Checklist S1 **CONSORT checklist.**
(DOCX)

Protocol S1 **Trial protocol.**
(DOCX)
