# Supplementary material for: Postoperative Neurocognitive Dysfunction in Patients Undergoing Cardiac Surgery after Remote Ischemic Preconditioning: A Double-Blind Randomized Controlled Pilot Study
Source: PLoS One. 2013 May 31;8(5):e64743. doi: 10.1371/journal.pone.0064743 (PMC3669352; doi:10.1371/journal.pone.0064743)
Supplement: File S1 — Supplemental methods. (DOCX) [file pone.0064743.s001.docx]

**FILE S1- Supplemental Methods**

All patients were visited and tested the day before surgery. Demographic data were analyzed, including education, medical history, and current medication. Signs and symptoms of depression were assessed preoperatively using the Beck depression inventory, and signs of dementia were investigated with the mini mental state examination (MMSE). Three months after surgery, patients were assessed for final neuropsychological evaluation.

Memory was evaluated with Rey’s auditory verbal learning test (RAVLT). The number of words recalled after the first, the second, and the third presentation of a 15-word list was summed up to determine a patient’s short-term memory (RAVLT 1-3). A free recall after 20 min was used to evaluate long-term memory (RAVLT LT). Three parallel versions of the word list were randomly used to minimize practice effects. Motor skills were evaluated with the Purdue pegboard test (PBT). The patients had to place pegs into the appropriate slots with their preferred hand (dominant) in the first run and with the non-preferred hand (non-dominant) in the second run. The required time was measured. Attention was tested with the Stroop color word interference test (STROOP). The patient was first asked to read aloud several lines of words that are names of colors (STROOP I). In part 2, the colors of patches are to be named (STROOP II), and in part 3, the interference section, the patient was instructed to state the color of ink used rather than the word: for example the word ‘‘red’’ may be printed in blue and so on (STROOP III). The patient was told to work as fast as possible. Within the trail making test (TMT), the patient had to connect consecutive numbers (part A) and to connect consecutive numbers and letters as fast as possible. The required time was measured. Patients were also tested with the Digit Span test (Digit span). First, the patients listened to a list of 3–9 digits and had to repeat them correctly. In the second run, the digits had to be repeated inverse to the listening order. The total number of correctly cited digits was measured. Finally, we performed the Digit symbol substitution test (DSST). Patients had to substitute defined symbols on a sheet of paper with numbers. The number of correctly substituted symbols in 90 s was counted and transformed into a score.

Executive functions were quantified with the verbal fluency test (VFT) including semantic and phonetic categories. These categories were pseudorandomized each time, choosing male and female first names, fruits/vegetables, and animals for semantic categories and different alphabetical letters for phonetic fluency. The sum of all runs was scored.
