# Supplementary material for: Postoperative Neurocognitive Dysfunction in Patients Undergoing Cardiac Surgery after Remote Ischemic Preconditioning: A Double-Blind Randomized Controlled Pilot Study
Source: PLoS One. 2013 May 31;8(5):e64743. doi: 10.1371/journal.pone.0064743 (PMC3669352; doi:10.1371/journal.pone.0064743)
Supplement: File S2 — Supplementary tables. Table S1, Reasons for missing neurocognitive assessment data. Table S2, Results of neurocognitive function assessed before, 5–7 days, and 3 months after surgery. Table S3, Neurocognitive decline/improvement in each neuropsychological test 5–7 days and 3 months after surgery compared with preoperative values. (DOCX) [file pone.0064743.s002.docx]

**File S2**

**Table S1. Reasons for missing neurocognitive assessment data**

|  | **After 5-7 days** | | **After 3 months** | |
| --- | --- | --- | --- | --- |
| **Variable** | **Control** | **RIPC** | **Control** | **RIPC** |
| **Total test battery not done** | n=29 | n=27 | n=23 | n=25 |
| Impaired MMSE (<24 points) | 4 | 5 | 3 | 2 |
| Refused assessment | 6 | 9 | 15 | 12 |
| Prolonged ICU therapy | 13 | 8 | - | - |
| Dead | - | - | 2 | 4 |
| Leaving the city | - | - | 1 | 4 |
| Others | 6 | 5 | 2 | 3 |
| **Sub-tests not done** | n=4 | n=9 | n=2 | n=7 |
| Red-green colour blind (STROOP test missing) | 1 | 5 | 1 | 5 |
| Postoperative upper limb paresthesia/ pain/ motoric impairment (PEGBOARD test missing) | 3 | 4 | 1 | 2 |

Data are presented as absolute number. No difference between groups. ICU indicates intensive care unit; MMSE, mini mental status examination; RIPC, remote ischemic preconditioning.

**Table S2. Results of neurocognitive function assessed before, 5-7 days, and 3 months after surgery**

|  | **Before surgery** | | **After 5-7 days** | | **After 3 months** | |
| --- | --- | --- | --- | --- | --- | --- |
| **Domains** | **Control** | **RIPC** | **Control** | **RIPC** | **Control** | **RIPC** |
| **MMSE** | 29 (24-30) | 29 (26-30) | 29 (24-30) | 29 (24-30) | 29 (26-30) | 30 (26-30) |
| **Memory** |  |  |  |  |  |  |
| **RAVLT 1-3 (n)** | 21 (6) | 21 (6) | 23 (6) | 22 (5) | 26 (7) | 24 (6) |
| **RAVLT LT (n)** | 6 (2-13) | 6 (0-14) | 8 (2-14) | 6 (3-13) | 9 (2-14) | 7 (2-15) |
| **Motor skills** |  |  |  |  |  |  |
| **PBT dominant (s)** | 85 (25-175) | 95 (60-235) | 91 (56-211) | 98 (64-213) | 77 (52-120) | 85 (58-147) |
| **PBT non-dominant (s)** | 90 (60-182) | 104 (63-273) | 98 (61-170) | 105 (73-182) | 85 (58-1219 | 98 (63-150) |
| **Attention** |  |  |  |  |  |  |
| **STROOP I (n)** | 12 (9-29) | 13 (8-36) | 13 (8-26) | 15 (10-20) | 12 (9-24) | 14 (9-23) |
| **STROOP II (n)** | 15 (10-34) | 16 (0-25) | 17 (11-45) | 20 (12-34) | 14 (9-27) | 16 (11-38) |
| **STROOP III (n)** | 35 (16-80) | 37 (18-91) | 36 (20-84) | 41 (14-87) | 30 (18-60) | 37 (14-86) |
| **TMT A (s)** | 34 (18-87) | 40 (13-95) | 32 (19-74) | 38 (20-62) | 32 (16-58) | 36 (19-58) |
| **TMT B (s)** | 87 (37-180) | 98 (36-218) | 82 (44-188) | 98 (32-180) | 75 (39-150) | 77 (46-162) |
| **Digit Span (n)** | 12 (4-21) | 11 (7-20) | 12 (6-19) | 11 (7-17) | 13 (6-19) | 12 (7-19) |
| **DSST (n)** | 37 (10) | 34 (10) | 36 (10) | 35 (11) | 42 (14) | 41 (10) |
| **Executive function** |  |  |  |  |  |  |
| **VFT semantic (n)** | 24 (7) | 22 (7) | 19 (4) | 17 (4) | 24 (7) | 25 (8) |
| **VFT phonetic (n)** | 11 (5) | 10 (4) | 11 (6) | 11 (4) | 10 (4) | 10 (4) |

Data are presented as median (range). No difference between groups.

RIPC indicates remote ischemic preconditioning; MMSE, mini mental status examination; RAVLT, rey’s auditorial verbal learning test first to third presentation of words (short-term memory); RAVLT LT, rey’s auditorial verbal learning test long-term memory; PBT dominant, purdue pegboard test performed with preferred hand; PBT non-dominant, purdue pegboard test performed with nonpreferred hand/other hand; STROOP, Stroop color word interference test, first to third run (I-III); TMT, trail making test part A and B; Digit, Digit Span test; DSST, digit symbol substitution test; VFT, verbal fluency test including semantic and phonetic categories (details of the test performance are described in Additional file 1).

**Table S3. Neurocognitive decline/improvement in each neuropsychological test 5-7 days and 3 months after surgery compared with preoperative values**

|  | **After 5-7 days** | | **After 3 months** | |
| --- | --- | --- | --- | --- |
| **Domains** | **Control** | **RIPC** | **Control** | **RIPC** |
| **Memory** |  |  |  |  |
| **RAVLT 1-3 (n)** | 8 / 13 | 7 / 15 | 4 / 22 | 4 / 19 |
| **RAVLT LT (n)** | 7 / 7 | 5 / 6 | 9 / 11 | 5 / 12 |
| **Motor skills** |  |  |  |  |
| **PBT dominant (s)** | 14 / 1 | 12 / 2 | 1 / 4 | 1 / 8 |
| **PBT non-dominant (s)** | 14 / 0 | 12 / 1 | 2 / 8 | 2 / 9 |
| **Attention** |  |  |  |  |
| **STROOP I (n)** | 7 / 10 | 4 / 11 | 7 / 9 | 7 / 6 |
| **STROOP II (n)** | 5 / 16 | 1 / 14 | 10 / 5 | 8 / 5 |
| **STROOP III (n)** | 2 / 3 | 5 / 10 | 6 / 2 | 9 / 9 |
| **TMT A (s)** | 9 / 7 | 12 / 2 | 11 / 7 | 10 / 3 |
| **TMT B (s)** | 10 / 4 | 11 / 4 | 5 / 5 | 3 / 7 |
| **Digit Span (n)** | 11 / 5 | 9 / 3 | 10 / 8 | 5 / 6 |
| **DSST (n)** | 5 /1 | 7 / 3 | 5 / 10 | 3 / 10 |
| **Executive function** |  |  |  |  |
| **VFT semantic (n)** | 32 / 1 | 21 / 1 | 16 / 11 | 11 / 12 |
| **VFT phonetic (n)** | 11 / 8 | 11 / 9 | 14 / 10 | 14 / 14 |

Data are presented as absolute number of patients. No difference between groups. A cognitive change was assumed if the preoperative to postoperative difference exceeded more than one SD.

RIPC indicates remote ischemic preconditioning; MMSE, mini mental status examination; RAVLT, rey’s auditorial verbal learning test first to third presentation of words (short-term memory); RAVLT LT, rey’s auditorial verbal learning test long-term memory; PBT dominant, purdue pegboard test performed with preferred hand; PBT non-dominant, purdue pegboard test performed with nonpreferred hand/other hand; STROOP, Stroop color word interference test, first to third run (I-III); TMT, trail making test part A and B; Digit, Digit Span test; DSST, digit symbol substitution test; VFT, verbal fluency test including semantic and phonetic categories (details of the test performance are described in Additional file 1).
