# Supplementary material for: Postoperative Neurocognitive Dysfunction in Patients Undergoing Cardiac Surgery after Remote Ischemic Preconditioning: A Double-Blind Randomized Controlled Pilot Study
Source: PLoS One. 2013 May 31;8(5):e64743. doi: 10.1371/journal.pone.0064743 (PMC3669352; doi:10.1371/journal.pone.0064743)
Supplement: Protocol S1 — Trial protocol. (DOCX) [file pone.0064743.s004.docx]

**Protocol S1: Trial Protocol**

# A. Title of Research Project

Remote Ischaemic Preconditioning for Heart Surgery- a randomized controlled trial **(RIPHeart-Study)**

**B. Area of Research:** Anaesthesiology

**C. Name of applicant**

Dr. med. Patrick Meybohm, MD

University Hospital Schleswig-Holstein, Campus Kiel, Department of Anaesthesiology and Intensive Care Medicine, Schwanenweg 21, D-24105 Kiel, Germany, Phone: +49 431 597-2991, Fax: +49 431 597-3002, e-mail: [meybohm@anaesthesie.uni-kiel.de](mailto:meybohm@anaesthesie.uni-kiel.de)

# D. Sponsoring institution/ mentor of the department

PD Dr. med. Berthold Bein, MD, DEEA

University Hospital Schleswig-Holstein, Campus Kiel, Department of Anaesthesiology and Intensive Care Medicine, Schwanenweg 21, D-24105 Kiel, Germany, Phone: +49 431 597-3739, Fax: +49 431 597-3002, e-mail: [bein@anaesthesie.uni-kiel.de](mailto:bein@anaesthesie.uni-kiel.de)

# E. Responsible financial officer

Prof. Dr. med. Jens Scholz, MD

University Hospital Schleswig-Holstein, Campus Kiel, Department of Anaesthesiology and Intensive Care Medicine, Schwanenweg 21, D-24105 Kiel, Germany, Phone: +49 431 597-2970, Fax: +49 431 597-3002, e-mail: [scholz@anaesthesie.uni-kiel.de](mailto:scholz@anaesthesie.uni-kiel.de)

**2. Research Summary**

Cardiac surgery with cardiopulmonary bypass is associated with a predictable incidence of myocardial and neurological dysfunction due to perioperative ischaemia that accounts for significant morbidity and mortality. Cardiac surgery is further associated with a substantial systemic inflammatory response and oxidative stress contributing to multi-organ dysfunction. Remote ischaemic preconditioning (RIPC) has been demonstrated as a novel, simple, non-invasive and inexpensive intervention in which brief ischaemia of non-vital tissue protects remote organs from a sustained episode of ischaemia. The purpose of this study is to evaluate the effects of RIPC prior to cardiopulmonary bypass on the incidence and severity of perioperative ischaemic events leading to myocardial and brain dysfunction in patients undergoing complex cardiac surgery compared to control intervention. The primary endpoint is postoperative neurocognitive dysfunction. Secondary endpoints are myocardial injury assessed by Troponin I, length of stay on the intensive care unit and total hospital stay. Furthermore, we investigate underlying pathways of RIPC in modifying the perioperative stress response with respect to myocardial tissue protein expression profile.

**3. Research Plan**

# 3.1. Introduction

# 3.1.1 Objectives

Cardiac surgery with cardiopulmonary bypass (CPB) is associated with a predictable incidence of myocardial and neurological dysfunction due to perioperative ischaemia that accounts for significant morbidity and mortality. Remote ischaemic preconditioning (RIPC) is a novel, simple, non-invasive and inexpensive intervention in which brief ischaemia of non-vital tissue protects remote organs from a sustained episode of ischaemia. The purpose of this study is to evaluate the effects of RIPC prior to CPB on the incidence and severity of perioperative ischaemic events leading to myocardial and brain dysfunction in patients undergoing complex cardiac surgery compared to control intervention.

**Key primary endpoint**

1. Postoperative neurocognitive dysfunction (POCD) after 5-7 days postoperative

**Key secondary endpoints**

1. Postoperative neurocognitive dysfunction (POCD) after 3 months
2. Incidence and severity of ischaemic events (myocardial infarction, instable angina, stroke) during hospital stay
3. Cardiac performance analysed by echocardiography
4. Cardiac performance analysed by cardiac MRI

6. Serum inflammatory biomarkers (CrP, cytokines, chemokines)

7. Myocardial tissue protein expression profile of heat shock protein-70, nitrous oxide synthetase, protein kinase C, mitogen-activated protein kinase (p38, ERK1/2), hypoxia inducible factor-1, and calcitonin gene-related protein

8. Transcriptional profiling with microarrays of leukocyte gene expression 24 hours after surgery.

**3.1.2 Background**

**Medical problem:** Cardiac surgery with CPB remains one of the most common surgical procedures, performed in about 1.5 million patients worldwide each year. Although surgical outcome has improved over time ^1^, increasingly older patients with multiple co-morbidities still remain at high risk for perioperative morbidity and mortality due to intraoperative impairment of vital organ perfusion and oxygenation. Moreover, cardiac surgery is generally associated with a predictable incidence of myocardial, neurological and renal dysfunction that accounts for significant morbidity and mortality of up to 10% depending on age, sex, co-morbidities and complexity of surgical procedures ^2-5^.

Postischaemic myocardial dysfunction after cardiac surgery is attributable to a multi-factorial aetiology, e.g. myocardial stunning, apoptosis, MI and ischaemia-reperfusion-induced injury. Clinically low cardiac output and hypotension requiring inotropic support are common findings. With respect to CABG alone, 16% of patients may experience myocardial infarction, severe ventricular dysfunction, heart failure, and/or death despite advances in surgical technique, CPB, and anaesthesia ^6^.

In addition, brain injury after CPB remains a common and serious complication ^7^. According to the ACC/AHA guidelines ^5^ postoperative neurological deficits can be classified into type 1 injuries, which are predominantly focal stroke, transient ischaemic attack, and fatal cerebral injuries in 3-5 % of patients due to macroembolism, and type 2 events, which reflect a more global/diffuse injury, with disorientation, agitation and postoperative neurocognitive dysfunction (POCD). POCD has been reported in 30 to 79% of patients at discharge and 24% to 57% at 6 months ^8^. Embolic events, changes in cerebral blood flow, global hypoperfusion, cerebral reperfusion injury, and a CPB-triggered whole body inflammatory response represent possible mechanisms for the high incidence of POCD. Elderly patients are more susceptible to cognitive impairment and stroke after cardiac surgery than others ^9^. Thus, with the increase in the percentage of elderly patients undergoing cardiac surgery, there has been a parallel increase in the incidence of stroke and POCD ^10^. Further, neurological injury negatively impacts the patient’s quality of life and health resources.

Finally, despite significant improvements in perioperative management, cardiac surgery is further associated with a substantial systemic inflammatory response and oxidative stress contributing to multi-organ dysfunction, morbidity and long-term mortality ^11^. These are triggered by the activation of leukocytes, complement, expression of adhesion molecules, cytokine release, and an increase in reactive oxygen species ^3^. Therefore, the development of strategies to control the inflammatory response continues to be the focus of extensive research.

**Remote ischaemic preconditioning (RIPC):** The most promising clinical examples for ischaemic preconditioning are angina preceding MI or transient ischaemic attacks preceding a full stroke; these preceding sub-lethal stressors are thought to enhance the tolerance of the organ to cope with the subsequent ischaemic event. However, the clinical applicability of local ischaemic preconditioning is limited by the need to induce ischaemia in the vulnerable target organ, a process that itself may aggravate organ injury and dysfunction. A more clinically relevant stimulus represents RIPC where ischaemia of non-vital tissue might protect remote organs against ischaemia-reperfusion injury, regardless of the trigger. This powerful, innate protection is mediated by recruitment of a neuronal pathway ^12^ and various neurohumoral factors such as adenosin, opioids, bradykinine, and calcitonin gene related protein ^13-16^. During the course of previous experimental investigations, brief ischaemia of the kidney, the intestine or skeletal muscle led to reduction of subsequent MI after prolonged coronary occlusion and protection against CPB-induced neural, pulmonary and myocardial damage ^17^. In humans, it has been shown that RIPC performed by transient limb ischaemia reduces ischaemia-induced endothelial dysfunction ^18^ and decreases markers of myocardial and renal injury in patients undergoing abdominal aortic aneurysm repair ^19^. In addition, RIPC modified expression of genes coding for key proteins involved in cytokine synthesis, chemotaxis, adhesion, innate immunity signalling pathways, and apoptosis in circulating human neutrophils ^20^. Finally, Hausenloy and colleagues demonstrated in a “proof-of-principle” study that RIPC reduced troponin T release by 43% as a surrogate parameter for long-term clinical outcome in the perioperative period in patients undergoing CABG ^21^. Thus, RIPC may represent an easily accessible, novel, non-invasive and inexpensive method to potentially reduce the incidence and severity of perioperative ischaemic events such as MI, stroke and ARF.

The rationale for studying RIPC in the setting of heart surgery is based on both the considerable experimental evidence that RIPC reduces myocardial ^14^ and neuronal ^22^, as wells as a few pilot trials ^19,21,23^. However, even when RIPC has been shown to reduce infarct size ^21^, this may not necessarily translate into a readily demonstrable neurological benefit, such as reduced POCD.

With respect to neurological injury, propofol and thiopental both have failed to alter outcome after cardiac surgery ^24,25^, and even therapies as potent as moderate hypothermia have been consistently shown to have little or no benefit concerning cognitive outcome ^26^. This indicates that the mechanism of CPB-induced neurological injury is more complex and cannot be sufficiently prevented with protective mechanisms offered by either hypothermia or anaesthetics alone. Thus, despite years of research, the treatment and prevention of ischaemic brain injury after cardiac surgery remains a major medical challenge. A novel method is warranted to prevent unfavourable events and to reduce POCD.

Therefore, we are now processing a prospective, randomised, blinded, single-centre study examining the effect of RIPC in patients undergoing complex heart surgery who have to deal with a predictable high incidence of myocardial and neurological injury and dysfunction that accounts for significant morbidity and mortality ^27^. Furthermore, investigating the role of RIPC in modifying the perioperative stress response at the genomic, molecular, cellular and tissue levels will improve scientific knowledge of underlying pathways.

So far, there have been no trials performed investigating RIPC in the course of complex heart surgery (CABG, aortic- or mitral valvular surgery, combined valvular and CABG surgery, and combination of ascending aortic aneurysm surgery, valvular or CABG surgery), and analysing its impact on patient’s neurological outcome. Therefore, this trial is needed now.

**3.1.3. Specific Aims**

**Major hypothesis:** RIPC results in less brain injury reflected by less neurocognitive dysfunction compared to control intervention after 5-7 days.

**Minor hypothesis:** RIPC results in less POCD compared to control intervention after 3 months, in less myocardial injury, better short-term outcome in terms of length of stay on the intensive care unit and total hospital stay. Further, we hypothesise that RIPC will modulate myocardial tissue protein expression profile of key proteins involved in organ protection in a subgroup of patients.

**3.2. Methods**

**3.2.1. Describe data collection techniques.**

**3.2.1.1. Co-workers**

-> Names in brackets indicate the role of each person involved in the project

- Dr. P. Meybohm (PM), PD Dr. med. Berthold Bein (BB), PD Dr. ret. nat. Martin Albrecht (MA), Prof. Dr. med. Jens Scholz (JS); Department of Anaesthesiology and Intensive Care Medicine, University Hospital Schleswig-Holstein, Campus Kiel, Germany
- Prof. J. Cremer (JC); Department of Heart and Vascular Surgery, University Hospital Schleswig-Holstein, Campus Kiel, Germany
- Prof. K. Zacharowski (KDZ); Department of Anaesthesiology and Intensive Care Medicine, University Frankfurt, Germany

**3.2.1.2. Clinical procedures (PM, BB, JS, JC):** One-hundred eighty patients undergoing cardiac surgery (single CABG, aortic- or mitral valvular surgery, combined valvular and CABG surgery, and combination of ascending aortic aneurysm surgery, valvular or CABG surgery) will be included. Thus, study population reflects a large group of patients with increased risk of perioperative morbidity and mortality. Following exclusions criteria will be defined: Emergency cases, myocardial infarction (MI) up to 7 days and stroke up to 2 months prior to enrolment, age below 18 years, off-pump heart surgery, renal failure, drug therapy with sulfonamide and nicorandil, ejection fraction less than 30%, and previous psychiatric and neurological illness. All patients will receive standard perioperative care. The applicant (PM) will inform the patient about the nature of the trial, its aims, expected advantages as well as possible risks. Each patient must consent in writing to participate in the study. The informed consent will be signed by both patient and treating investigator.

Patients will be randomly assigned to receive either control intervention or RIPC before CPB. RIPC will be induced during anaesthesia by four 5-min cycles of right upper limb ischaemia and 5-min reperfusion using a blood-pressure cuff inflated to 200 mmHg (at least to a pressure 15 mm Hg greater than the systolic arterial pressure) measured via the arterial line ^17,23,28^. Control patients undergo sham placement of the blood pressure cuff around the upper limb with inflation to 20 mmHg. Upper limb ischaemia provided by blood-pressure cuff inflation will be performed by a single study assistant, and will be blinded to the anaesthesia team, cardiac surgeon and the patient. The anaesthesia team will perform standardised anaesthesia in all patients.

Midazolam 3.75-7.5 mg will be given orally to every patient 1 h before surgery. On arrival in the anaesthetic room, a peripheral venous and an arterial cannula will be inserted before anaesthesia. Anaesthesia will be induced with propofol (2 mg/kg), sufentanil (0.1 μg/kg), and rocuronium (0.6 mg/kg). The trachea will be intubated and mechanical ventilation will be started with oxygen with room air to achieve an end-tidal carbon dioxide tension of 4–5 kPa. Before cardiopulmonary bypass, anaesthesia will be maintained with propofol (≈ 4-6 mg/kg/h) and sufentanil (≈ 0.5-1.5 µg/kg/h). A 7F saline filled catheter will be inserted in the right internal jugular vein for determination of central venous pressure and drug administration. Arterial blood pressure, leads I and II of the electrocardiogram, and nasopharyngeal temperature will be recorded continuously. Standard CPB techniques with blood cardioplegia will be used.

Transesophageal echocardiographic variables (E/A-Ratio, left ventricular ejection fraction) will be subsequently obtained at baseline, after RIPC, and after 15 and 30 min after CPB (BB). Cardiac MRI is aimed to be performed preoperative, 5-7 days and 3 months postoperative to analyze right and left ventricular performance ^29,30^.

All patients will undergo a battery of 10 neuropsychological tests 1 day before, 5-7 days and 3 months after surgery by a blinded study nurse. In accordance with the statement of consensus on assessment of neurobehavioral outcome after cardiac surgery ^31^, the battery will include tests for motor skills, verbal memory capacity, and attention. In addition, tests will be included to assess speed and capacity of working memory, visuospatial capacity, selective and sustained attention, and information processing. Cognitive decline will be defined as a decrease in an individual’s performance of at least 20% from baseline, in at least 20% of the main variables ^32^.

**3.2.1.3. Determination of biochemical plasma markers (PM, BB, MA, KDZ):** Arterial blood samples will be collected at baseline, after CPB, and after 6, 12, 24 and 48 hours after surgery. Plasma will be obtained (centrifuged at 3000g for 5 min) and stored at -20°C until assayed. In a blinded fashion, cardiac troponin I, total creatine kinase, and inflammatory biomarkers (Cytokine multiPlex for Luminex laser; Invitrogen, Karlsruhe, Germany) will be determined using the microsphere array technique (Luminex 100 system; Luminex, Austin, USA) as previously described ^33^.

**3.2.1.4. Determination of leukocyte gene expression (MA):** In a subgroup of fifty patients (n=25 of each group), exemplarily, transcriptional profiling of leukocyte gene expression before and 24 hours after surgery will be analysed by microarrays. Blood samples will be collected into PAXgene tubes for immediate mRNA stabilization and extraction. Different gene-expression pathways involving inflammation, cellular adhesion, apoptosis and stress response will be analysed ^20,34^. Experiments will be performed in collaboration with the Institute of Clinical Molecular Biology, Kiel, Germany, using an in-house research microarray according to the guidelines of the Microarray Gene Expression Data (MGED) society ^35^.

**3.2.1.5. Determination of myocardial tissue expression profile (MA):** In a subgroup of sixty patients, exemplarily, myocardial tissue from the right atrium will be collected before cannula insertion and at the end of CPB, and stored at -20°C until assayed. Gene and protein expression profile of different mediators known to play an important role in preconditioning mechanisms, such as heat shock protein-70, nitrous oxide synthetase, protein kinase C, mitogen-activated protein kinase (p38, ERK1/2), hypoxia inducible factor-1, and calcitonin gene-related protein will be determined by standard Western blotting and real-time RT-PCR.

**3.2.2. Describe types of data to be obtained and statistical or power analysis if indicated**

The sample size calculation is based on the assumptions that the incidence of cognitive decline at 5-7 days postoperative is 30% after on-pump surgery ^36-38^, and that one-third reduction could be achieved using RIPC. Individual patients may decline intra-hospital study follow up. We estimated loss to follow up as 5 % of the patients based on previous experience for clinical studies in our hospital. A total of 90 patients per group were estimated.

Secondary endpoints will be analysed in an exploratory way by standard statistical methods for comparing independent samples with respect to their measurement level and/or type of distribution (parametric and nonparametric methods).

With respect to feasibility of recruitment, the intended recruitment rate is based on a prospective recording of all potentially included patients from October to December 2008 in our institution. Each week a mean of eight patients fulfilling the inclusion criteria could be included. Thirty percent of these patients were estimated to give informed consent regarding study participation corresponding to two to three patients per week. Based on 52 weeks of recruitment per year and one study team (one anaesthetist, one nurse) available we calculated a realistic enrolment of 140 patients per year.

**3.2.3. Point out potential problems and limitations**

Including patients with a full range of cardiac surgery procedures (e.g. CABG, aortic- or mitral valvular surgery, combined valvular and CABG surgery, and combination of ascending aortic aneurysm surgery, valvular or CABG surgery) might result in heterogeneous data with high variance. However, our proposed study population reflects a large group of patients with increased risk of perioperative morbidity and mortality.

**3.2.4. Statement of approval**

This study will be performed in accordance with the revision of the Declaration of Helsinki (1996). Study protocol, patient information and informed consent will be send to the ethics committee of the University of Kiel and the competent authorities for appraisal.

##### 3.3. References

1. Ferguson TB, Hammill BG, Peterson ED, DeLong ER, Grover FL. A decade of change-risk profiles and outcomes for isolated coronary artery bypass grafting procedures, 1990-1999: a report from the STS National Database Committee and the Duke Clinical Research Institute. Ann Thorac Surg 2002;73:480-9.

2. Newman MF, Wolman R, Kanchuger M, Marschall K, Mora-Mangano C, Roach G, Smith LR, Aggarwal A, Nussmeier N, Herskowitz A, Mangano DT. Multicenter preoperative stroke risk index for patients undergoing coronary artery bypass graft surgery. Multicenter Study of Perioperative Ischemia (McSPI) Research Group. Circulation 1996;94:II74-80.

3. Paparella D, Mickleborough LL, Carson S, Ivanov J. Mild to moderate mitral regurgitation in patients undergoing coronary bypass grafting: effects on operative mortality and long-term significance. Ann Thorac Surg 2003;76:1094-100.

4. Lu JC, Shaw M, Grayson AD, Poullis M, Pullan M, Fabri BM. Do beating heart techniques applied to combined valve and graft operations reduce myocardial damage? Interact Cardiovasc Thorac Surg 2008;7:111-5.

5. Eagle KA, Guyton RA, Davidoff R, Edwards FH, Ewy GA, Gardner TJ, Hart JC, Herrmann HC, Hillis LD, Hutter AM, Jr., Lytle BW, Marlow RA, Nugent WC, Orszulak TA. ACC/AHA 2004 guideline update for coronary artery bypass graft surgery: a report of the American College of Cardiology/American Heart Association Task Force on Practice Guidelines. Circulation 2004;110:e340-437.

6. Mangano DT, Tudor IC, Dietzel C. The risk associated with aprotinin in cardiac surgery. N Engl J Med 2006;354:353-65.

7. Arrowsmith JE, Grocott HP, Reves JG, Newman MF. Central nervous system complications of cardiac surgery. Br J Anaesth 2000;84:378-93.

8. Baumgartner WA. Neurologic injury after cardiopulmonary bypass surgery. J Neurosurg Anesthesiol 2004;16:102-4.

9. Peterson ED, Cowper PA, Jollis JG, Bebchuk JD, DeLong ER, Muhlbaier LH, Mark DB, Pryor DB. Outcomes of coronary artery bypass graft surgery in 24,461 patients aged 80 years or older. Circulation 1995;92:II85-91.

10. Mohan R, Amsel BJ, Walter PJ. Coronary artery bypass grafting in the elderly--a review of studies on patients older than 64, 69 or 74 years. Cardiology 1992;80:215-25.

11. De Hert SG. Outcome after cardiovascular surgery: where do we stand? Curr Opin Anaesthesiol 2008;21:47-9.

12. Loukogeorgakis SP, Panagiotidou AT, Broadhead MW, Donald A, Deanfield JE, MacAllister RJ. Remote ischemic preconditioning provides early and late protection against endothelial ischemia-reperfusion injury in humans: role of the autonomic nervous system. J Am Coll Cardiol 2005;46:450-6.

13. Addison PD, Neligan PC, Ashrafpour H, Khan A, Zhong A, Moses M, Forrest CR, Pang CY. Noninvasive remote ischemic preconditioning for global protection of skeletal muscle against infarction. Am J Physiol Heart Circ Physiol 2003;285:H1435-43.

14. Heidbreder M, Naumann A, Tempel K, Dominiak P, Dendorfer A. Remote vs. ischaemic preconditioning: the differential role of mitogen-activated protein kinase pathways. Cardiovasc Res 2008;78:108-15.

15. Hausenloy DJ, Yellon DM. Remote ischaemic preconditioning: underlying mechanisms and clinical application. Cardiovasc Res 2008.

16. Wolfrum S, Nienstedt J, Heidbreder M, Schneider K, Dominiak P, Dendorfer A. Calcitonin gene related peptide mediates cardioprotection by remote preconditioning. Regul Pept 2005;127:217-24.

17. Kharbanda RK, Li J, Konstantinov IE, Cheung MM, White PA, Frndova H, Stokoe J, Cox P, Vogel M, Van Arsdell G, MacAllister R, Redington AN. Remote ischaemic preconditioning protects against cardiopulmonary bypass-induced tissue injury: a preclinical study. Heart 2006;92:1506-11.

18. Loukogeorgakis SP, Williams R, Panagiotidou AT, Kolvekar SK, Donald A, Cole TJ, Yellon DM, Deanfield JE, MacAllister RJ. Transient limb ischemia induces remote preconditioning and remote postconditioning in humans by a K(ATP)-channel dependent mechanism. Circulation 2007;116:1386-95.

19. Ali ZA, Callaghan CJ, Lim E, Ali AA, Nouraei SA, Akthar AM, Boyle JR, Varty K, Kharbanda RK, Dutka DP, Gaunt ME. Remote ischemic preconditioning reduces myocardial and renal injury after elective abdominal aortic aneurysm repair: a randomized controlled trial. Circulation 2007;116:I98-105.

20. Konstantinov IE, Arab S, Kharbanda RK, Li J, Cheung MM, Cherepanov V, Downey GP, Liu PP, Cukerman E, Coles JG, Redington AN. The remote ischemic preconditioning stimulus modifies inflammatory gene expression in humans. Physiol Genomics 2004;19:143-50.

21. Hausenloy DJ, Mwamure PK, Venugopal V, Harris J, Barnard M, Grundy E, Ashley E, Vichare S, Di Salvo C, Kolvekar S, Hayward M, Keogh B, MacAllister RJ, Yellon DM. Effect of remote ischaemic preconditioning on myocardial injury in patients undergoing coronary artery bypass graft surgery: a randomised controlled trial. Lancet 2007;370:575-9.

22. Ren C, Gao X, Steinberg GK, Zhao H. Limb remote-preconditioning protects against focal ischemia in rats and contradicts the dogma of therapeutic time windows for preconditioning. Neuroscience 2008;151:1099-103.

23. Cheung MM, Kharbanda RK, Konstantinov IE, Shimizu M, Frndova H, Li J, Holtby HM, Cox PN, Smallhorn JF, Van Arsdell GS, Redington AN. Randomized controlled trial of the effects of remote ischemic preconditioning on children undergoing cardiac surgery: first clinical application in humans. J Am Coll Cardiol 2006;47:2277-82.

24. Zaidan JR, Klochany A, Martin WM, Ziegler JS, Harless DM, Andrews RB. Effect of thiopental on neurologic outcome following coronary artery bypass grafting. Anesthesiology 1991;74:406-11.

25. Roach GW, Newman MF, Murkin JM, Martzke J, Ruskin A, Li J, Guo A, Wisniewski A, Mangano DT. Ineffectiveness of burst suppression therapy in mitigating perioperative cerebrovascular dysfunction. Multicenter Study of Perioperative Ischemia (McSPI) Research Group. Anesthesiology 1999;90:1255-64.

26. Grigore AM, Mathew J, Grocott HP, Reves JG, Blumenthal JA, White WD, Smith PK, Jones RH, Kirchner JL, Mark DB, Newman MF. Prospective randomized trial of normothermic versus hypothermic cardiopulmonary bypass on cognitive function after coronary artery bypass graft surgery. Anesthesiology 2001;95:1110-9.

27. Bech-Hanssen O, Ryden T, Schersten H, Oden A, Nilsson F, Jeppsson A. Mortality after mitral regurgitation surgery: importance of clinical and echocardiographic variables. Eur J Cardiothorac Surg 2003;24:723-30.

28. Schmidt MR, Smerup M, Konstantinov IE, Shimizu M, Li J, Cheung M, White PA, Kristiansen SB, Sorensen K, Dzavik V, Redington AN, Kharbanda RK. Intermittent peripheral tissue ischemia during coronary ischemia reduces myocardial infarction through a KATP-dependent mechanism: first demonstration of remote ischemic perconditioning. Am J Physiol Heart Circ Physiol 2007;292:H1883-90.

29. Selvanayagam JB, Petersen SE, Francis JM, Robson MD, Kardos A, Neubauer S, Taggart DP. Effects of off-pump versus on-pump coronary surgery on reversible and irreversible myocardial injury: a randomized trial using cardiovascular magnetic resonance imaging and biochemical markers. Circulation 2004;109:345-50.

30. Pegg TJ, Selvanayagam JB, Karamitsos TD, Arnold RJ, Francis JM, Neubauer S, Taggart DP. Effects of off-pump versus on-pump coronary artery bypass grafting on early and late right ventricular function. Circulation 2008;117:2202-10.

31. Murkin JM, Newman SP, Stump DA, Blumenthal JA. Statement of consensus on assessment of neurobehavioral outcomes after cardiac surgery. Ann Thorac Surg 1995;59:1289-95.

32. Stump DA. Selection and clinical significance of neuropsychologic tests. Ann Thorac Surg 1995;59:1340-4.

33. Zacharowski K, Zacharowski PA, Koch A, Baban A, Tran N, Berkels R, Papewalis C, Schulze-Osthoff K, Knuefermann P, Zahringer U, Schumann RR, Rettori V, McCann SM, Bornstein SR. Toll-like receptor 4 plays a crucial role in the immune-adrenal response to systemic inflammatory response syndrome. Proc Natl Acad Sci U S A 2006;103:6392-7.

34. Seeburger J, Hoffmann J, Wendel HP, Ziemer G, Aebert H. Gene expression changes in leukocytes during cardiopulmonary bypass are dependent on circuit coating. Circulation 2005;112:I224-8.

35. Brazma A, Hingamp P, Quackenbush J, Sherlock G, Spellman P, Stoeckert C, Aach J, Ansorge W, Ball CA, Causton HC, Gaasterland T, Glenisson P, Holstege FC, Kim IF, Markowitz V, Matese JC, Parkinson H, Robinson A, Sarkans U, Schulze-Kremer S, Stewart J, Taylor R, Vilo J, Vingron M. Minimum information about a microarray experiment (MIAME)-toward standards for microarray data. Nat Genet 2001;29:365-71.

36. Van Dijk D, Jansen EW, Hijman R, Nierich AP, Diephuis JC, Moons KG, Lahpor JR, Borst C, Keizer AM, Nathoe HM, Grobbee DE, De Jaegere PP, Kalkman CJ. Cognitive outcome after off-pump and on-pump coronary artery bypass graft surgery: a randomized trial. Jama 2002;287:1405-12.

37. Newman MF, Kirchner JL, Phillips-Bute B, Gaver V, Grocott H, Jones RH, Mark DB, Reves JG, Blumenthal JA. Longitudinal assessment of neurocognitive function after coronary-artery bypass surgery. N Engl J Med 2001;344:395-402.

38. Hernandez F, Jr., Brown JR, Likosky DS, Clough RA, Hess AL, Roth RM, Ross CS, Whited CM, O'Connor GT, Klemperer JD. Neurocognitive outcomes of off-pump versus on-pump coronary artery bypass: a prospective randomized controlled trial. Ann Thorac Surg 2007;84:1897-903.

**Changes of the study plan (date 01/01/2009)**

Due to technical limitations, lack of financial resources and lack of human resources the following endpoints will not be investigated:

- Cardiac performance analysed by echocardiography
- Cardiac performance analysed by cardiac MRI
- Serum inflammatory biomarkers (CrP, cytokines, chemokines)
- Transcriptional profiling with microarrays of leukocyte gene expression 24 hours after surgery
